# Occult HCV or delayed viral clearance from lymphocytes of Chronic HCV genotype 3 patients after interferon therapy

**DOI:** 10.1186/1479-0556-9-14

**Published:** 2011-09-06

**Authors:** Ambreen G Muazzam, Saleem Qureshi, Atika Mansoor, Lubna Ali, Musarrat Iqbal, Saima Siddiqi, Khalid M Khan, Kehkashan Mazhar

**Affiliations:** 1 Institute of Biomedical and Genetic Engineering, Islamabad, Pakistan; 2 Dept of Medicine, KRL General Hospital, Islamabad, Pakistan; 3 Pir Mehr Ali Shah Arid Agriculture University, Rawalpindi, Pakistan

## Abstract

**Background:**

A recently discovered occult HCV entity reported by various investigators seems to be highly controversial. Especially, the clinical significance of these findings remains uncertain. For optimal outcome of antiviral therapy, investigation of occult HCV needs a broad-based probe in order to investigate the results of viral therapy and its host/viral interaction. The current study was aimed at determining the prevalence of occult HCV in peripheral blood lymphocytes of predominantly genotype 3 HCV-infected patients after completion of antiviral therapy and to investigate long term outcomes in the presence or absence of PBMC positivity.

**Method:**

A total of 151 chronic, antiHCV and serum RNA-positive patients were enrolled in the study. Patients with a complete virological response at the end of treatment were screened for the presence of viral RNA in their PBMCs and were followed for up to one year for the presence of serum and PBMC viral genomic RNA.

**Results:**

Out of 151 patients, 104 (70%) responded to the prescribed interferon treatment and showed viral-clearance from serum. These were screened for the presence of genomic RNA in their PBMCs. Sixteen samples were PBMC-positive for viral RNA at the end of treatment (EOT). All these patients had also cleared the virus from peripheral blood cells after the 6-12 month follow-up study.

**Conclusion:**

True occult hepatitis C virus does not exist in our cohort. Residual viremia at the EOT stage merely reflects a difference in viral kinetics in various compartments that remains a target of immune response even after the end of antiviral therapy and is eventually cleared out at the sustained viral response (SVR).

## Introduction

Hepatitis C is the leading cause of liver disease infecting about 200 million individuals worldwide. HCV is a single-stranded virus of the flaviviridae family that replicates by its negative strand. In most cases of infection (85%) the virus evades the immune system and establishes a chronic infection that may lead to cirrhosis and liver carcinoma [1,2]. The hallmarks of chronic infection are antiHCV positivity, presence of genomic HCV RNA in the serum for more than six months and abnormal liver function tests. Current treatment standards with pegylated interferon alpha (PEG IFN-alpha) and ribavirin in genotype 1 and PEG/Standard Interferon with Ribavirin in genotype 2,3 result in sustained response rates of 50% and 76-80% respectively [2,3]. In addition to the large number of non-responding patients the treatment has further limitations due to its toxicity and high cost, especially for patients in the developing world. Moreover, recent data suggest that response rates in genotype 3 are not as optimal as previously believed with relapse rates up to 40% resulting in a growing pool of patients who fail to clear the virus [4]. Attempts to improve response rates have focused on pretreatment and treatment predictors like viral kinetics in an attempt to optimize treatment duration and improve sustained outcome. Eradication of the virus is judged by the absence of viral RNA from the serum with normalization of liver function tests. PCR-based methods that can detect very low levels of viral genome have given a new dimension to the analysis of treatment response and monitoring of the later consequences of the disease.

Occult HCV infection, a recently identified type of HCV infection that is still controversial is defined as the persistent presence of detectable HCV RNA in hepatocytes or peripheral blood mononuclear cells (PBMCs) of patients with undetectable plasma HCV-RNA by conventional PCR assays [5,6]. It is thought that the presence of occult-HCV may be indicated by abnormal liver function tests or antiHCV positivity although the virus may persist for years after spontaneous recovery or after sustained viral response (SVR) [6]. Hepatic cells are the primary targets of the virus. Occult is usually more established in hepatocytes but lymphocytes have also been found to serve as a 'hideout'. Some investigators have also reported the presence of intermediate negative strands in the PBMC, thereby indicating active viral replication and persistence in PBMCs. The identification of occult was made possible due to the higher sensitivity of PCR-based methods [7-9]. It is believed that the immune system and liver tissue act as reservoirs of this kind of infection resulting in relapse and potential return to overt infectious state. In addition, it is believed that occult RNA can lead to persistent minimal inflammation or even chronic active hepatitis. Occult hepatitis C is considered a milder disease than chronic hepatitis C but little is known about clinical history, progression or outcomes [5]. Occult HCV infection has also been identified in chronic hepatitis C patients who have responded to antiviral therapy with sero-clearance and normalization of liver functions. This finding therefore challenges the belief that serum HCV resolution reflects complete viral eradication.

In Pakistan, there are approximately 10 million people living with hepatitis C infection [10]. Current study was undertaken in order to determine the prevalence of occult HCV among predominantly geno 3-infected chronic hepatitis C patients responding to antiviral therapy and to analyze its effect on the final outcomes. Due to higher cost, PEG interferon is given as second line therapy only to patients who do not respond to Standard Interferon Therapy (SIT). Liver biopsy samples are considered the gold standard for occult HCV RNA detection, but performing such invasive procedure is considered unethical, especially in the absence of any clinical presentation. Because PBMC analyses have proved to be equally useful for this purpose, we therefore opted for the noninvasive PBMC analyses in order to determine the presence occult HCV in our cohort [9]. The cohort consisted of patients who had attained a complete end-treatment response. In order to detect the occult phase at an early stage we assumed that PBMC positivity at EOT might reflect a higher prevalence of the occult HCV. The study is an effort to add information to the complex natural history of HCV disease which shows interplay of the host, virus and the environment.

## Materials and methods

**Figure 1:**
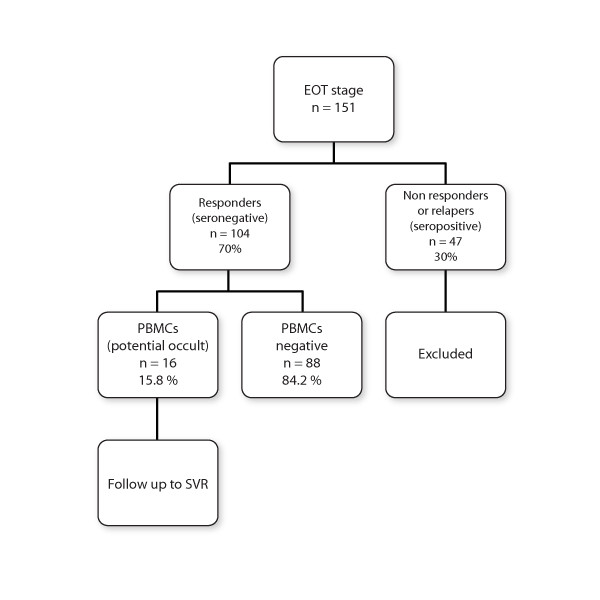
**Study design**.

The patient cohort included nonalcoholic, chronic liver disease patients from the Dept. of Medicine, KRL General Hospital, Islamabad, Pakistan. Most patients were residents of the Potohar region who visited the hospital during 2007-2009 and who reached the EOT stage of antiviral therapy. Exclusion criteria were immunocompromised patients including pregnant women, HIV-positive patients, patients on steroids and patients who were HBV-positive or positive for any other viral infections. The study was conducted according to ethical guide lines of the 2000 Helsinki Declaration, and duly approved by the ethical committee of the institute. Patients gave written informed consent for their participation in the study. Plan of the study is summarized in Figure 1, briefly, blood samples of the patients (151) were collected at the end of treatment (EOT) and serum was screened for viral RNA. Serum-negative patients were selected (104) and their PBMCs isolated subsequently. The patients were divided into two groups. The first group consisted of treatment-naïve patients (n = 87) who were administered Standard Interferon Therapy with Ribavirin for 72 weeks and the second group (n = 17) included treatment-experienced patients who were administered PEG interferon with Ribavirin for 24 weeks as the second line of treatment. SIT/ribavirin is the standard method of treatment in this region due to the high cost of PEG interferon which is administered only as second line therapy to the approximately 30% patients who remain non SVR [11]. Table 1 shows the base-line parameters of the patients. Same patients were followed at SVR (6-12 months) and their PBMCs isolated again and analyzed for the presence of genomic HCV RNA.

**Table 1:** Patients baseline parameters

	Group 1	Group 2
	Naive(n = 87)	Experienced(n = 17)

**Gender (Male:Female)**	30:57	3:14

**Age (mean ± SD)**	42.9 ± 10.6	47.29 ± 8.92

**ALTs (IU/ml ± SD)**	74.8 ± 60.1	94.1 ± 78.8

**BMI (± SD)**	23.88 ± 6.6	24.64 ± 5.2

**Cholesterol (± SD)**	181.42 ± 38.2	188.07 ± 22.9

**Triglycerides (± SD)**	177.16 ± 53.22	170.15 ± 50.03

**Base line Quant (range)**	1 × 10^3^-5.8 × 10^6^	1 × 10^3^-2 × 10^6^

**LDL (normal/high)**	58/4	13/0

**HDL(normal/high)**	58/4	13/0

**Inflammation**		

**A1**	38 (.43)	9 (.53)

**A2**	5 (.057)	1 (.058)

**A3**	15 (.17)	5 (.294)

**Not done**	29 (.33)	2 (.11)

**Fibrosis**		

**F0**	45(.52)	10(.59)

**F1**	13(.15)	3(.17)

**F2**	5(.005)	1(.005)

**F3**	1(.001)	0

**Not done**	23(.26)	3(.17)

A1, A2, A3 are mild, moderate and marked inflammation respectively

F0, F1, F2, F3 are no, mild, moderate and marked fibrosis respectively

### HCV-RNA detection

HCV RNA was detected by using a commercially available RT-PCR kit COBAS AMPLICOR Hepatitis C virus test ver 2.0 (Roche Molecular Diagnostics, Mannheim, Germany) with a sensitivity limit of 50 IU/ml and 99% specificity. Briefly, viral RNA was extracted from plasma by lysis of viral particles with guanidinium thiocyanate (chaotropic agent), followed by alcohol precipitation. HCV RNA was retrotranscribed to cDNA and amplified by the single tube RT-PCR primer set, KY78 (5'-CTCGCAAGCACCCTATCAGGCAGT-3') and KY80 (5'-GCAGAAAGCGTCTAGCCATGGCGT-3'), to amplify a sequence of 244 nucleotides within the conserved 5'UTR of the HCV genome. Amplified DNA was detected using target-specific oligonucleotide probes that permitted independent identification of HCV amplicons and internal control amplicons.

### Isolation of serum and PBMC

Serum was separated from whole blood in vacutainers containing serum enhancer following centrifugation. Isolated serum was immediately stored at -20°C in appropriate aliquots in order to avoid repeat freeze-thawing. Peripheral blood lymphocytes were isolated immediately following blood drawing (5.0 ml) from serum-negative patients. Whole blood was layered over Ficoll-Hypaque (Sigma, USA) density- gradient medium. Cells were isolated from the buffy coat after centrifugation and were washed three times with phosphate buffered saline (pH 7.0). The cells were observed under inverted microscope and counted using a haemacytometer. Aliquots of approximately a million cells suspended in RNAse-free dd H_2_O were then preserved at -70°C to avoid repeat freeze/thawing. HCV RNA was analyzed from cell lysates using the Roche Amplicor kit. Two positive control samples from the blood of serum positive patients were processed along with the batch of test samples.

### Statistical analyses

Analyses of base line parameters with PBMC-positive and -negative patients were performed using SPSS ver 10.0 for windows (SPSS, Inc; Chicago, IL, USA).

## Results

One hundred and fifty one patients were evaluated after the end of prescribed antiviral therapy. Out of these, 104 patients (70%) showed treatment response by clearing the virus from the serum, while 47 patients were either relapsers or non-responders to the prescribed therapy at the end of treatment. From the responders, 87 were treatment-naïve and 17 were treatment experienced patients. All the responders were tested for the presence of virus in their peripheral blood mononucleocytes. Sixteen patients (15.8%) were found positive for the viral RNA, 11 from the naïve and 5 from the treatment-experienced patients (Table 2). These patients were considered occult and we applied the 2 × 2 contingency chi-square test to determine if there was any association with occult HCV in the two groups of patients, i.e treatment-naïve and treatment-experienced. No significant association was found (Pearson chi square = 0.08). We also applied the chi square as well as one way ANOVA to work out association between all the basic parameters listed in Table 1 for both groups of patients and did not find any significant associations. All these patients were followed up for SVR and their PBMC were analyzed at the SVR stage as well. All patients who showed positivity at the end of treatment, cleared the virus at the SVR stage. We also observed one lymphocyte sample that remained slightly positive after 6 months but cleared the virus completely by 12 months post-EOT.

**Table 2:** Baseline parameters of all patients who were serum negative, PBMC positive at the EOT stage

**Ser No**.	Gender	Age yrs	ALT	cholest	HDL	LDL	T4	Treat	Quant x10^6^	Inflm	Fibr
**1**	M	35	NA	NA	NA	NA	NA	SIT	.56	3	3

**2**	M	55	18	NA	NA	NA	NA	SIT	1	3	2

**3**	F	34	71	90	40	102	178	SIT	.13	1	1

**4**	M	48	93	178	30	129	188	SIT	.006	3	0

**5**	F	32	78	199	38	96	179	SIT	.046	1	0

**6**	F	39	87	207	37	125	273	SIT	.001	1	0

**7**	F	49	76	160	42	98	180	SIT	1	NA	0

**8**	F	40	19	200	36	98	120	SIT	1	1	0

**9**	M	60	88	180	42	90	78	SIT	.0063	3	2

**10**	F	52	50	236	32	90	140	SIT	1	1	0

**11**	F	44	60	139	36	96	144	SIT	NA	1	0

**12**	F	37	96	190	36	107	220	SIT^r^	1.81	1	1

**13**	F	36	19	139	40	75	140	SIT^r^	.3	1	0

**14**	F	62	62	201	34	102	178	SIT^r^	.04	1	0

**15**	F	49	46	NA	NA	NA	NA	SIT^r^	.0127	NA	NA

**16**	F	51	112	198	32	86	120	SIT^nr^	2.13	3	0

SIT^r ^relapse to SIT

SIT^nr ^nonresponder to SIT

NA not available

## Discussion

Occult HCV infection has been defined as the presence of HCV RNA in liver and/or lymphoid cells despite undetectable HCV-RNA in the serum in HCV-infected patients who have a spontaneous clearance or antiviral treatment response. The current study was primarily aimed at determining the prevalence of occult HCV in predominantly 3(a/b)-infected HCV patients and secondly to investigate if PBMC positivity can be seen as an indicative marker for later recurrence of viral particles in the serum. Our cohort was infected with the subtype of occult HCV defined by the presence of HCV RNA in PBMCs of patients with treatment-induced HCV RNA clearance from serum (antiHCV- positive, serum HCV RNA-negative). It should also be mentioned that the sample collection was random for the genotype selection, but the cohort turned out to be 100% genotype 3, which has already been reported as the predominant type of HCV in Pakistan, especially in the Potohar region [11,12]. This also reinforces our own previous observation of genotype 3 as the most prevalent genotype in the region; therefore our conclusions are more relevant for HCV genotype 3. Our study also shows a 70% success rate for the sustained response achieved through interferon alpha/ribavirin combined therapy as reported previously [2] for genotype 3.

Liver biopsy samples are considered the gold standard for occult HCV diagnosis; but due to the invasive nature of this method, especially in the absence of clinical manifestations, reliable alternative methods of investigation have also been developed [7]. HCV RNA has been reported in PBMC as well as ultracentrifuged serum samples in a high percentage of patients (70%) and is considered a reliable method for occult detection as repeat liver biopsy samples are not usually available in clinical practice [13]. We therefore, relied on the HCV detection in lymphocytes for our method of investigation. According to the current definition of occult as serum-clear, treated patients, we considered the presence of positive PBMCs in the end-of-treatment specimens as evidence of occult HCV. Our results showed viral genomic HCV present in the PBMCs of 15.8% of 3 (a/b) chronically infected HCV patients at EOT. The follow up studies, however, showed that the HCV particles that are present in the PBMC of the patients at the end of treatment are ultimately cleared out by 6-12 months after EOT at the SVR stage. We did not find any association of delayed clearance with any of the baseline parameters listed in Table 1 as previously reported [14,15]. The SVR stage results therefore lead us to conclude that the 15.8% EOT viral presence reflects a difference in the viral kinetics in various compartments rather than a true case of occult HCV. It is also worth mentioning that there has been a recent report on the study of occult virus in the region; but the cohort consisted of non-treated cryptogenic virus-infected patients who were antiHCV-negative and showed increased ALT (alanine amino transferase) and AST (aspartate amino transferase) levels [16]. These results may reflect a true case of occult HCV. Our results also indicate the likelihood that the immune response remains active even after the completion of the treatment regimen and that it helps in clearing of the residual virions that might remain detectable in other compartments.

It could be argued that lack of detection of HCV in the PBMC of our cohort at SVR is due to low sensitivity of our test (50 IU/ml), which may be too low to detect the presence of very small amounts of occult viral particles; but in our study plan we followed the same cohort of patients who initially (EOT) showed the presence of occult virus in their PBMC with the same sensitivity level. It is also worth mentioning that one of the 'occult' samples, although still weakly positive at 6 months post EOT, was observed to gradually clear the residual viremia at one year post-treatment. However, we could not exclude the possibility of viral persistence in the hepatic cells in our cohort of patients as none of them presented any indication for a liver biopsy test, such as raised ALTs at the EOT. These patients will be periodically followed up and liver biopsies carried out if needed. According to our current findings we conclude that clinicians may be confident of the clearance of the virus as indicated by the absence of viral RNA in the serum at the EOT especially in the case of genotype 3 infection. A previous study on five geno 3 patients also showed clearance of virus after 56 months of treatment [17].

The question remaining is why the virus is cleared later from the PBMC than the serum (15.8% cases). This cannot be explained clearly due to the incomplete understanding of the HCV infection and evasion processes. It can be propounded, however, that HCV has developed a number of evasion mechanisms, infection of PBMCs being one of those where the virus can avoid the immune defense system, while hepatocytes remain the actual target. We tried to find an association of baseline viral load and other clinical parameters with final clearance of virus, since some researchers have shown an association of occult presence with high cholesterol and triglyceride levels [14,15]. Statistical tests were applied to determine if there was an association with any of the base line parameters given in Table 1, but no such evidence was found. It may also be that the virus developed some new quasi-species in PBMC that showed delayed response to the antiviral therapy but we could not confirm that speculation.

The outcome of therapy involves an interplay of host and viral factors in their specific environments, which ultimately determines the immune response at both the innate and humoral response levels of the host. It is difficult, therefore, at this stage to determine the effect of clinical and biochemical parameters as markers for disease outcome. It would be worthwhile to study the known host and viral genetic factors that might contribute to the immune response in the patients and provide a better understanding of the observed treatment responses. The existence of occult HCV as a reservoir of the virus, that can become active has remained controversial since its first report in 2004 [5]. There have been recent negative reports in other genotypes also [18-22]. Therefore, the picture is still unclear. Further detailed, long-term studies are needed before clinicians can be confident that the serum based tests are accurate.

## Conclusion

In conclusion, the results of our study show differences in viral kinetics in different compartments. Viral persistence in the PBMC compartment at the EOT stage and its subsequent clearance at the SVR stage shows delayed clearance in the lymphoid cells as compared to plasma. Persistence at the EOT stage is not an indicator of subsequent relapse by SVR stage.

## Competing interests

The authors declare that they have no competing interests.

## Authors' contributions

AGM, AM, LA and SS performed the experimental work and data analysis; SQ and MI helped in clinical investigations and contributed towards report writing; KM and KMK prepared the manuscript. All authors read and approved the final manuscript.
